# Intraspecific variation in leaf (poly)phenolic content of a southern hemisphere beech (*Nothofagus antarctica*) growing under different environmental conditions

**DOI:** 10.1038/s41598-024-69939-7

**Published:** 2024-08-29

**Authors:** M. Gabriela Mattera, Marina Gonzalez-Polo, Pablo L. Peri, Diego A. Moreno

**Affiliations:** 1 Institute of Forestry and Agricultural Research of Bariloche-IFAB (INTA Bariloche-CONICET), S.C. Bariloche, Argentina; 2 Institute for Biodiversity and Environment Research-INIBIOMA (CONICET-UNCo), S.C. Bariloche, Argentina; 3 National Institute of Agricultural Technology (INTA), National University of Patagonia Austral (UNPA), National Council for Scientific and Technical Research (CONICET), Santa Cruz, Argentina; 4 Phytochemistry and Health Food Laboratory (LabFAS), Center for Soil Science and Applied Biology of Segura (CEBAS-CSIC), Murcia, Spain

**Keywords:** Patagonian Ñire, Non-timber products, Chemical profiling, Species-local environment interaction, Plant sciences, Natural variation in plants, Plant ecology, Plant stress responses, Secondary metabolism

## Abstract

*Nothofagus antarctica* (G.Forst.) Oerst. (Ñire) leaves are a valuable source of (poly)phenolic compounds and represent a high-value non-timber product from Patagonian forests. However, information on the variability of their chemical profile is limited or non-existent. The aim of this study was to evaluate the (poly)phenolic variability in Ñire leaf infusions. To this end, different tree populations growing under different temperature regimes and soil characteristics were considered. Interestingly, a cup of Ñire leaf infusion could be considered as a rich source of quercetin. Significant differences in the (poly)phenolic content, especially in flavonoid conjugates and cinnamic acids, were found among the populations studied. These results suggest metabolic variability among the forests studied, which could be related to the species response to its growing conditions, and also provide some clues about the performance of *N. antarctica* under future climate scenarios. The *N. antarctica* forests growing in environments with lower frequency of cold and heat stress and high soil fertility showed better infusion quality. This study showed how a South American beech interacts with its local environment at the level of secondary metabolism. In addition, the information obtained is useful for defining forest management strategies in the Patagonian region.

## Introduction

(Poly)phenols are bioactive compounds of high relevance due to their known health benefits. Antioxidant, anti-inflammatory, anti-mutagenic and anti-carcinogenic properties are some of the most well-documented effects of these compounds^1–3^. In addition, several studies suggest that long-term (poly)phenolic intake protects against cardiovascular and neurodegenerative diseases, cancer, osteoporosis, and diabetes^4–6^. For these reasons, polyphenol-enriched products have attracted great interest from the food industry under the context of current consumer demands. In plants, these compounds are involved in the secondary metabolism and contribute to the plant tolerance against abiotic and biotic stresses^7,8^. (Poly)phenols are found widely in the fruits, vegetables, cereals and beverages and their concentration can be influenced by environmental growing conditions (such as temperature extremes, soil characteristics, sun exposure and drought), agronomic management, pest infestation and maturity at harvest, among others^9–11^. In addition, post-harvest processes and storage conditions can also affect the concentration of bioactive compounds in foods^9^. Currently, there is an increased interest in identifying some low-cost strategies to obtain plants with high concentrations of metabolites with potential benefits for human health^12^.

The South American ecosystems, such as the Patagonian region, constitute under-exploited sources of valuable bioactive compounds. Patagonia is a marginal region with an extension of 780.000 km^2^, characterised by extreme climate conditions^13^. Only 1% of the Patagonia area are irrigated valleys, which are associated with crop production^13^. The species *N. antarctica*, known as Ñire, is a tree native to the temperate forests of Patagonia and is one of the most widespread and cold-tolerant southern beeches. Its natural distribution range covers 751,640 ha in Argentina^14^, mostly along the Andean Mountain range. This species grows in a wide environmental range^15^, displaying a variety of morphotypes related to the growing conditions^16^. However, its current range could be altered by the effects of climate change in the Patagonian region. A general warming and an increase of the extreme temperatures have been predicted for this region in the 21st century^17,18^. A significant decrease in precipitation has also been predicted for northern Patagonia^18^. Most of the *N. antarctica* forests in Patagonia (~ 70%) are used under a silvopastoral system^19^, which is an efficient productive alternative in the region. In the last years, the "Manejo del Bosque con Ganadería Integrada (MBGI)" (Forest Management with Integrated Livestock) plan has been implemented, based on all the provisioning services offered by *N. antarctica* forests and on the sustainable management that takes into account the conservation of species. Under this management, *N. antarctica* species constitutes a relevant source of timber (mainly poles, firewood and timber for rural construction^20^) and non-timber products^21,22^ which are important for increasing the profitability of the system. In folk medicine, leaves and young buds of Ñire have been used as a febrifuge^23^. Hikers and climbers use its leaves to elaborate infusions in mountain huts^24^. The organoleptic and nutraceutical properties of *N. antarctica* infusions have been previously highlighted, such as their pleasant aroma and high phenolic content^25^. Terpenoids and flavonoid glycosides and aglycones were found in the lipophilic exudate of its young leaves^26^. In addition, *N. antarctica* leaf infusion has been suggested as a valuable source of (poly)phenolic compounds and its combination with green tea may increases its health benefits by increasing (poly)phenolic content and antioxidant capacity^22^. For these reasons, *N. antarctica* leaves and young buds are high-value non-timber products of interest to the food and pharmaceutical industries, with a positive impact on regional economies. However, information on the quality differences of *N. antarctica* leaves as a raw material for industry is very limited or non-existent. Thus, the present study aimed to evaluate the chemical variability of *N. antarctica* leaf infusions, considering different tree populations growing under different temperature regimes and soil characteristics. The results of this study would be useful for identifying outstanding forests as a source of superior non-timber products for industry, and for supporting management programs for this species. In addition, the information about the differential response of *N. antarctica* forests at the level of secondary metabolism is key to understanding the performance of this species under future climate scenarios.

## Results

### Characterization of the population sites

The environmental characterization of the sites where *N. antarctica* populations were sampled is summarised in Table 1. Since the temperature dataset did not exhibit a normal distribution, medians were chosen for describing this variable. During two growing seasons corresponding to September 2020 to August 2022, differences in air temperature regimes were found among the sampled sites. In the hottest month (i.e., January), the forest site that is located at 41º 25ʹ and 880 m a.s.l. (*LG_up 50m*) showed higher median maximum temperature and lower median minimum temperature than the two northern sites (*LF_lake level* and *LF_up 50m*). Furthermore, the frequency (%) of days with temperatures up to 30 °C was 0.08 in the *LG_up 50m*, being significantly higher than in the other three sites (0.04 in *LG_lake level*, 0.03 in *LF_up 50m* and 0.02 in *LF_lake level*) (Supplementary Table 1). In the coldest month (i.e., July), no differences in the median maximum temperature were found among the sampled sites, but both southern (*LG*) locations showed a lower median minimum temperature than northern (*LF*) ones. In addition, both *LG* sites showed frequencies of days with temperature below 0 °C ranging between 0.36 and 0.30%, which were significantly higher than the frequencies in the northern (*LF*) sites (ranging between 0.23 and 0.22%) (Supplementary Table 2). The frequencies of days with temperatures below – 5 °C were also significantly higher in *LG* locations (ranging between 0.06 and 0.03%) than in *LF*s (0.01–0.008%, respectively) (Supplementary Table 2). These findings showed that southern populations experienced more extreme growing conditions during the study period, with higher frequencies of day with temperatures inducing abiotic stresses (e.g., dehydration stress due to heat and frost). In addition, the site where the *LG_up 50m* population grows also showed a higher daily thermal range in the hottest month compared to the other sites (Supplementary Table 3).

**Table 1:** Characterization of studied sites considering two growing seasons.

ID	Latitude	Longitude	Altitude	Air temperature in the hottest month (January)		Air temperature in the coldest month (July)		
				Tª max	Tª min	Tª max	Tª min	
*LF_lake level*	40° 26ʹ (LF)	71° 32ʹ	931 (lake level)	22.67 B	6.72 A	7.28 A	− 0.94 B	
*LF_up 50m*	40° 26ʹ (LF)	71° 32ʹ	985 (up 50 m)	23.58 B	7.03 A	6.57 A	− 0.94 B	
*LG_lake level*	41° 25ʹ (LG)	71° 29ʹ	830 (lake level)	24.79 AB	4.68 B	7.73 A	− 1.85 A	
*LG_up 50m*	41° 25ʹ (LG)	71° 29ʹ	880 (up 50 m)	27.86 A	5.45 B	7.98 A	− 1.11 A	

ID	Soil features							
	pH	EC (dS m^−1^)	Ni (mg kg^−1^)	Am (mg kg^−1^)	Total C (g kg^−1^)	Total N (g kg^−1^)	P-Olsen (mg kg^−1^)	pNmin (mg kg^−1^)
*LF_lake level*	6.04 ± 0.20 a	0.10 ± 0.04 a	6.98 ± 2.34 a	3.37 ± 1.22 a	36.39 ± 5.06 a	2.22 ± 0.47 a	2.60 ± 0.58 a	53.10 ± 7.52 a
*LF_up 50m*	5.85 ± 0.08 a	0.08 ± 0.02 a	9.59 ± 4.30 a	4.66 ± 1.78 a	64.49 ± 1.18 a	4.28 ± 0.09 a	1.14 ± 0.16 a	47.00 ± 4.94 a
*LG_lake level*	5.90 ± 0.02 a	0.05 ± 0.01 b	4.84 ± 1.51 a	4.39 ± 0.93 a	66.04 ± 12.78 a	4.01 ± 0.64 a	1.58 ± 0.30 a	27.60 ± 5.07 b
*LG_up 50m*	5.94 ± 0.09 a	0.04 ± 0.01 b	4.78 ± 2.52 a	1.58 ± 0.63 a	40.32 ± 8.42 a	3.29 ± 0.77 a	1.37 ± 0.36 a	32.90 ± 4.22 b

*LF*: Falkner Lake; *LG*: Guillelmo Lake. Tª max: median of maximum air temperature; Tª min: median of minimum air temperature; EC: electric conductivity; Ni: nitrate (N-NO_3_^−^) concentration; Am: ammonium (N-NH_4_^+^) concentration; total N and total C: nitrogen (N) or carbon (C) concentration; P-Olsen: concentration of the extractable phosphorus; pNmin: potential N mineralization after 16 weeks of incubation. All soil variables are shown as mean ± standard error. Lower-case letters refer to an ANOVA test and cupper-cases letters refer to a non-parametric test. In the same column, different letters mean that exist significant differences (*p-value* < 0.05).

At the soil level, the northern (*LF*) forests seem to have a higher level of fertility than the southern forests. This fact is evidenced by the higher electrical conductivity and potential mineralization rate in *LF* sites compared to *LG*s (Table 1). Soils from *LF* sites have the potential to mineralize approximately 40% more organic nitrogen into inorganic forms (ammonium and nitrate), making this nitrogen available to plants, compared to soils from *LG* sites. Potential mineralization tests were carried out under optimal conditions of soil moisture and temperature, which can be considered as indicators of the Nitrogen fractions readily available to nitrifiers and thus, of the amount of available N that the soil can potentially release to the plants. In addition, both latitudinal sites (*LF* and *LG*) had phosphorous (P) concentrations (i.e., Olsen-P in mg kg^−1^) that are below the recommended critical value for plant P availability (< 10 mg Olsen-P per kg soil; Table 1). Regarding total C and N concentrations in the studied sites, there is an interaction between latitude and altitude factors and thus, no clear trend was observed.

### (Poly)phenolic content in the *N. antarctica* populations

Significant differences in the total (poly)phenolic content and total flavonoid derivatives (*p-value* < 0.05) were found among *N. antarctica* infusions (i.e., raw material from different populations) (Table 2). At the level of individual compound, the concentration of DiHHDPg, DiGHHDPg, Tretragalloyl-glucose, Caffeoyl hexoside, 3-Caffeoyl quinic acid, 3-*p*-Coumaroyl quinic acid, Quercetin-hexoside, Quercetin-pentoside, Quercetin-rhamnoside, and Quercetin-Galloyl-pentoside were significantly different among populations.

**Table 2:** Polyphenol concentration for each *N. antarctica* populations.

ID	AOV				mg/100 ml of infusion							
	Lat (*LF*, *LG*)	Alt (*lake level*, *up 50m*)	Lat x Alt	ID	*LF_lake level*		*LF_up 50m*		*LG_lake level*		*LG_up 50m*	
DiHHDPg	n.s.	n.s.	*	0.020*	7.58 ± 0.51	a	11.50 ± 0.62	a	10.22 ± 0.26	a	1.32 ± 1.41	b
DiGHHDPg	*	n.s.	n.s.	0.020*	1.33 ± 0.06	a	0.51 ± 1.41	a	n.d	b	0.90 ± 0.22	a
TGg	n.s.	n.s.	*	0.012*	3.19 ± 0.32	ab	3.55 ± 0.37	a	1.91 ± 0.04	c	2.05 ± 0.00	bc
Ch	n.s.	n.s.	*	0.007*	4.83 ± 0.63	a	2.49 ± 0.46	b	0.53 ± 1.41	c	3.85 ± 0.21	ab
X3CQA	n.s.	n.s.	*	0.020*	8.88 ± 0.87	b	5.81 ± 0.07	b	8.98 ± 0.11	b	15.37 ± 0.14	a
X3pCoumQA	*	n.s.	n.s.	0.033*	4.69 ± 0.64	a	5.10 ± 0.40	a	1.86 ± 0.33	b	2.05 ± 0.49	b
X5CQA	n.s.	n.s.	n.s.	Non param.	0.29 ± 1.41	n.s.	0.3 ± 1.41	n.s.	0.38 ± 1.41	n.s.	n.d	n.s.
Mh	n.s.	n.s.	*	0.095	2.48 ± 0.14	n.s.	2.99 ± 0.10	n.s.	3.57 ± 0.35	n.s.	2.59 ± 0.16	n.s.
Mp	n.s.	n.s.	n.s.	0.340	3.94 ± 0.30	n.s.	3.17 ± 0.17	n.s.	3.40 ± 0.15	n.s.	3.67 ± 0.09	n.s.
QGh	n.s.	n.s.	*	Non param.	2.45 ± 0.19	n.s.	2.76 ± 0.15	n.s.	4.90 ± 0.29	n.s.	2.98 ± 0.02	n.s.
Qh	n.s.	n.s.	*	0.000*	20.00 ± 0.01	a	18.49 ± 0.02	b	16.31 ± 0.09	c	10.68 ± 0.03	d
Qp	*	*	n.s.	0.009*	9.62 ± 0.10	a	9.27 ± 0.00	a	9.32 ± 0.22	a	6.77 ± 0.04	b
Qr	n.s.	n.s.	n.s.	0.0153*	3.49 ± 0.59	n.s.	5.36 ± 0.13	n.s.	4.10 ± 0.29	n.s.	4.30 ± 0.09	n.s.
QGp	*	*	n.s.	0.004*	2.64 ± 0.22	a	2.34 ± 0.19	a	2.22 ± 0.29	a	0.66 ± 1.41	b
Gd	*	n.s.	n.s.	0.015*	16.94 ± 0.02	a	18.04 ± 0.44	a	12.66 ± 0.15	ab	8.12 ± 0.30	b
Qd	n.s.	n.s.	*	0.029*	13.86 ± 0.37	ab	11.21 ± 0.26	b	11.22 ± 0.19	b	17.42 ± 0.06	a
CF	n.s.	n.s.	*	0.003*	44.62 ± 0.05	a	44.39 ± 0.01	a	43.82 ± 0.14	a	31.65 ± 0.01	b
TP	*	n.s.	n.s.	0.009*	75.41 ± 0.09	a	73.64 ± 0.14	a	67.70 ± 0.10	a	57.18 ± 0.06	b

*LF_lake level*, *LF_up 50m*, *LG_lake level*, *LG_up 50 m*: population IDs following Table 1. Lat: latitude, Alt: altitude, Lat x Alt: latitude-altitude interaction. HHDP: hexahydroxydephenic acid. DiHHDPg: Di-HHDP-glucoside, DiGHHDPg: Di-Galloyl-HHDP-glucoside, TGg: Tetragalloyl-glucose, Ch: Caffeoyl-hexoside:, X3CQA: 3-Caffeoyl quinic acid, X3pCoumQA: 3-p-Coumaroyl-QA, X5CQA: 5-Caffeoyl quinic acid, Mh: Myricetin-hexoside, Mp: Myricetin-pentoside, QGh: Quercetin-Galloyl-hexoside, Qh: Quercetin-hexoside, Qp: Quercetin-pentoside, Qr: Quercetin-rhamnoside, QGp: Quercetin-Galloyl-pentoside, Gd: Gallic acid derivatives, Qd: Quinic acid derivatives, CF: Conjugated Flavonoids , TP: Total (Poly)phenolic content. *: differences were significant (p-value < 0.05); AOV: analysis of variance considering Lat, Alt or ID as main factor as well as the Lat x Alt interaction; non-param.: a non-parametric method was used because the variable does not have a normal distribution. Letters correspond to a post-hoc test *(p-value* < 0.05) after AOV. In the same row, different letters indicate significant differences; n.s.: not significant. n.d.: not detected.

**Figure 1:**
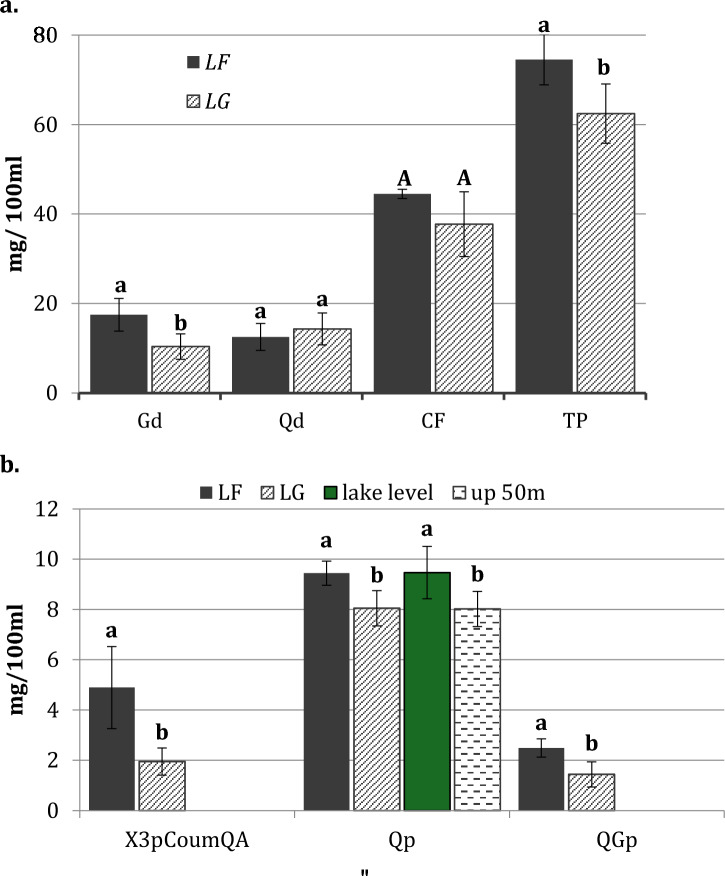
Concentration (mg/100 ml of infusion) of the (poly)phenols showing significant differences among *N. antarctica* populations: (**a**) differences in the Gallic acid derivatives (Gd), Quinic acid derivatives (Qd), Conjugated Flavonoids (CF), and Total (Poly)phenolic content (TP); (**b**) differences in some individual (poly)phenolic compounds (X3pCoumQA: 3-p-Coumaroyl-QA, Qp: Quercetin-pentoside, and QGp: Quercetin-Galloyl-pentoside). The intervals in each bar correspond to the standard error. Statistical analysis was performed within each (poly)phenolic group/compound. Lower-case letters correspond to an ANOVA analysis and upper-case letters correspond to a non-parametric test.

When latitude and altitude variables were considered as the main factors in the analysis of variance, most of the individual compounds showed significant latitude-altitude interactions (Table 2). Among the variables where the interaction was not significant, total (poly)phenolic content and Gallic acid derivatives were significantly higher in the northern (*LF*) populations (Fig. 1a). Similar results were obtained for the concentration of DiGHHDPg, 3-p-Coumaroyl quinic acid, Quercetin-pentoside and Quercetin-Galloyl-pentoside compounds (Fig. 1b). In addition, significant differences were found between altitudes (*lake level vs up 50m*) for both Quercetin- pentoside and Quercetin-Galloyl-pentoside compounds (Fig. 1b), which were significantly higher in the lake level (*LF_lake level* and *LG_lake level*) populations.

**Figure 2:**
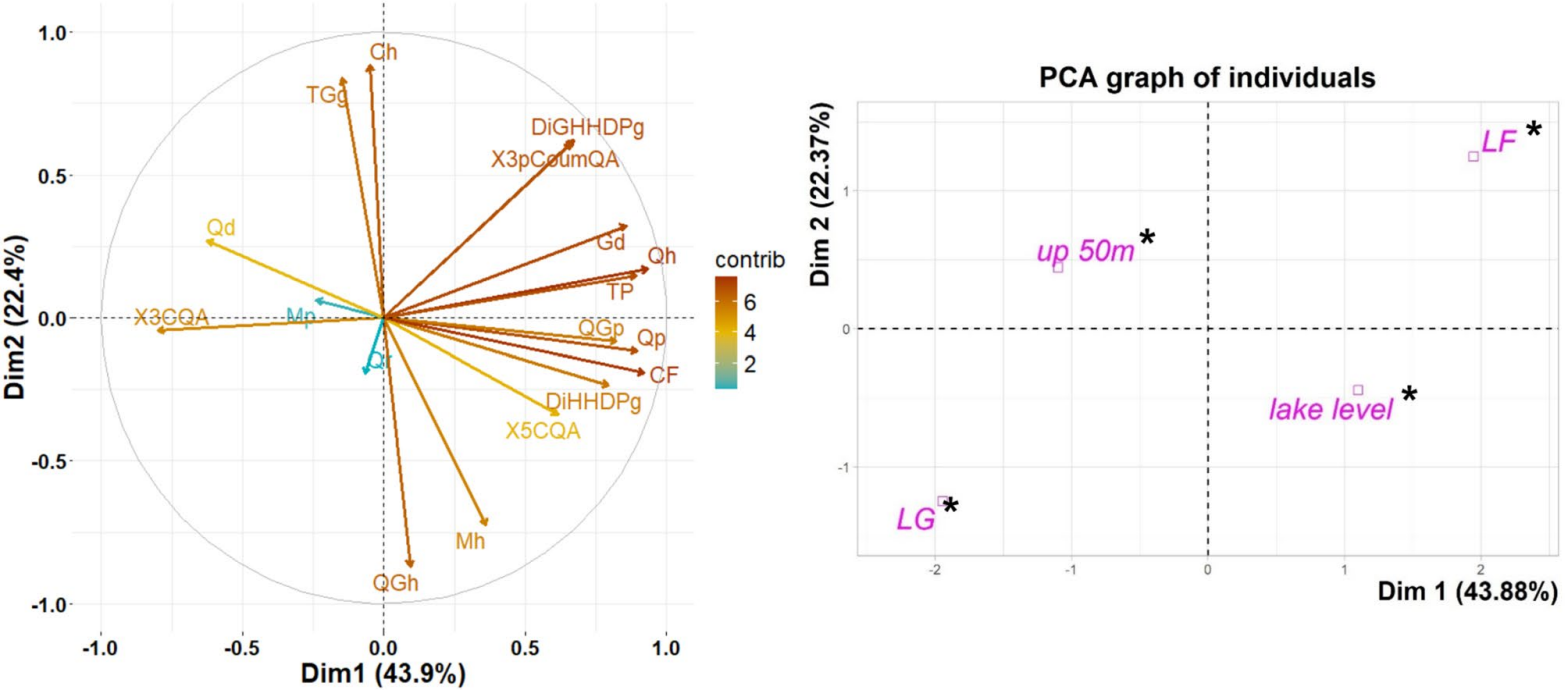
Principal Component Analysis (PCA) of the (poly)phenolic concentration in the infusions elaborated with *N. antarctica* leaves from different populations. (**a**) PCA of (poly)phenolic compounds considering their contributions to dimensions 1 and 2; (**b**) PCA of individuals grouped by supplementary variables (i.e., means of latitude and altitude). HHDP: hexahydroxydephenic acid. DiHHDPg: Di-HHDP-glucoside, DiGHHDPg: Di-Galloyl-HHDP-glucoside, TGg: Tetragalloyl-glucose, Ch: Caffeoyl-hexoside:, X3CQA: 3-Caffeoyl quinic acid, X3pCoumQA: 3-p-Coumaroyl-QA, X5CQA: 5-Caffeoyl quinic acid, Mh: Myricetin-hexoside, Mp: Myricetin-pentoside, QGh: Quercetin-Galloyl-hexoside, Qh: Quercetin-hexoside, Qp: Quercetin-pentoside, Qr: Quercetin-rhamnoside, Gd: Gallic acid derivatives, Qd: Quinic acid derivatives, CF: Conjugated Flavonoids, TP: Total (Poly)phenolic content. *Lat.LF*: populations located at 40° 26ʹS, *Lat.LG:* populations located at 41° 25ʹS, *lake level*: populations located at 931 and 830 m a.s.l.; *up 50m*: populations located at 985 and 880 m a.s.l. The colours of the arrows correspond to the contribution scale of the right bar. *Significant contribution of the supplementary variables to each dimension by *v*-test.

The Principal Component Analysis of the (poly)phenolic concentrations in all *N. antarctica* infusions showed that two first principal components (PC1 and PC2) explained the 66.25% of the accumulate variance among populations (Fig. 2) and PC3 and PC4 together explained another 23.46% of the found variance (data not shown).

**Figure 3:**
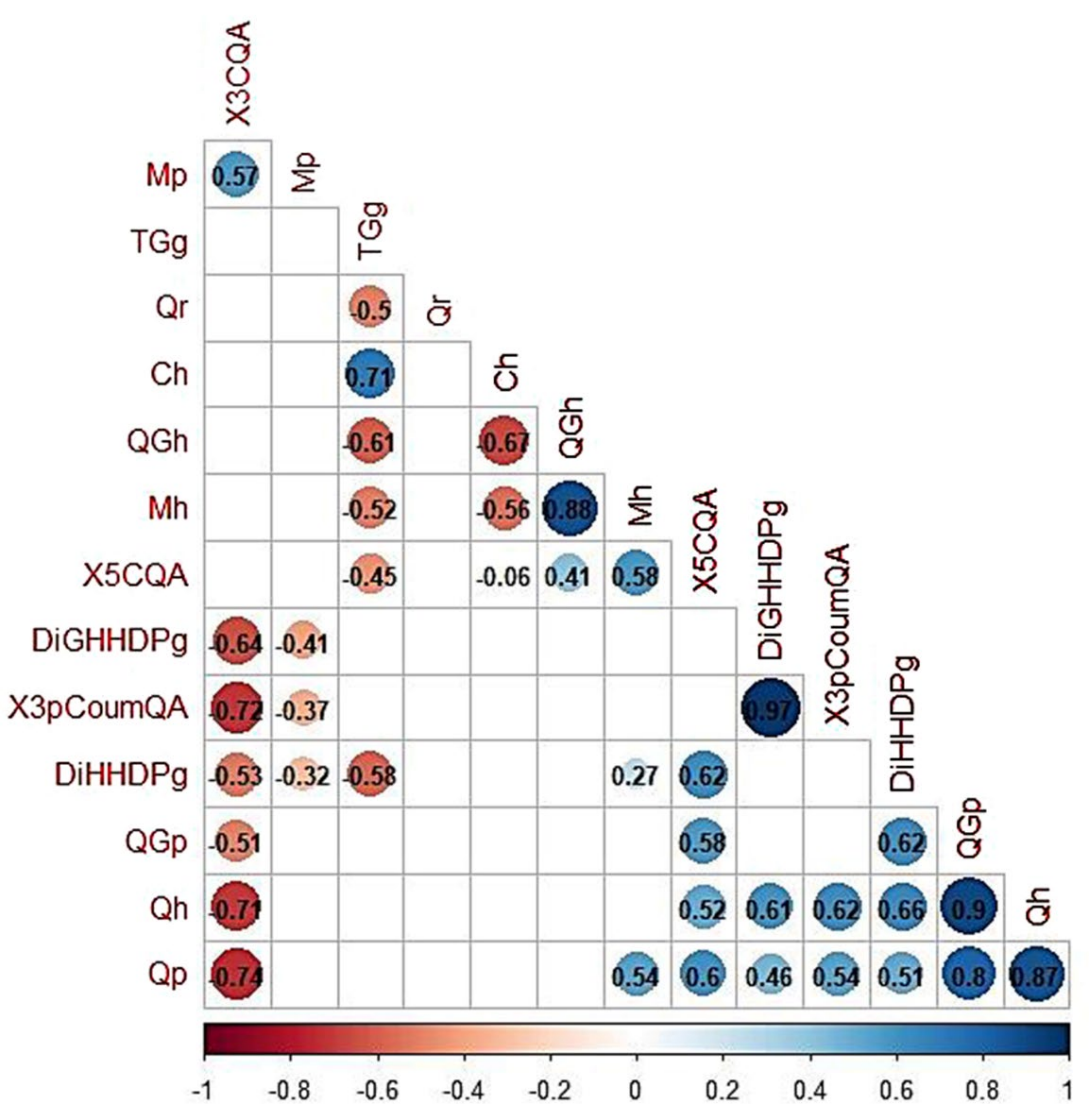
Correlation analysis (“spearman rank coefficient”) among (poly)phenolic compounds. Only the values of significantly correlated compounds are shown. Abbreviations correspond to the compound names that are mentioned in Fig. 1.

Among all variables, Quercetin -hexoside, Quercetin -pentoside, Conjugated Flavonoids, and Total (Poly)phenols together contributed more than 42.05% to the first dimension (Supplementary Fig. 1). On the other hand, Tetragalloyl-glucose, Quercetin-Galloyl-hexoside and the Caffeoyl-hexoside contributed in more than 55.70% to the second dimension. Regarding the supplementary variables, latitude significantly contributed to the explained variation given by dimension 1, whereas altitude significantly contributed to the explained variation given by dimension 2. In addition, twelve pairs of compounds showed a positive and strong (≥ 0.6 correlation coefficient) correlation (Fig. 3), and six pairs are negatively and strongly correlated (≤ -0.6 correlation coefficient). 3-Caffeoyl quinic acid was negatively correlated to 3-p-Coumaroyl quinic acid, Quercetin-pentoside, Quercetin-hexoside, and Di-Galloyl-HHDP-glucose (correlation coefficient of -0.72,  -0.74,  -0.71 and  -0.64, respectively). A new PCA was performed considering only one of the variables from each pair of highly correlated groups (higher than 0.8) and the results obtained were similar to the previous ones (data not shown).

### (Poly)Phenolics and soil features in the studied *N. antarctica* forests

**Figure 4:**
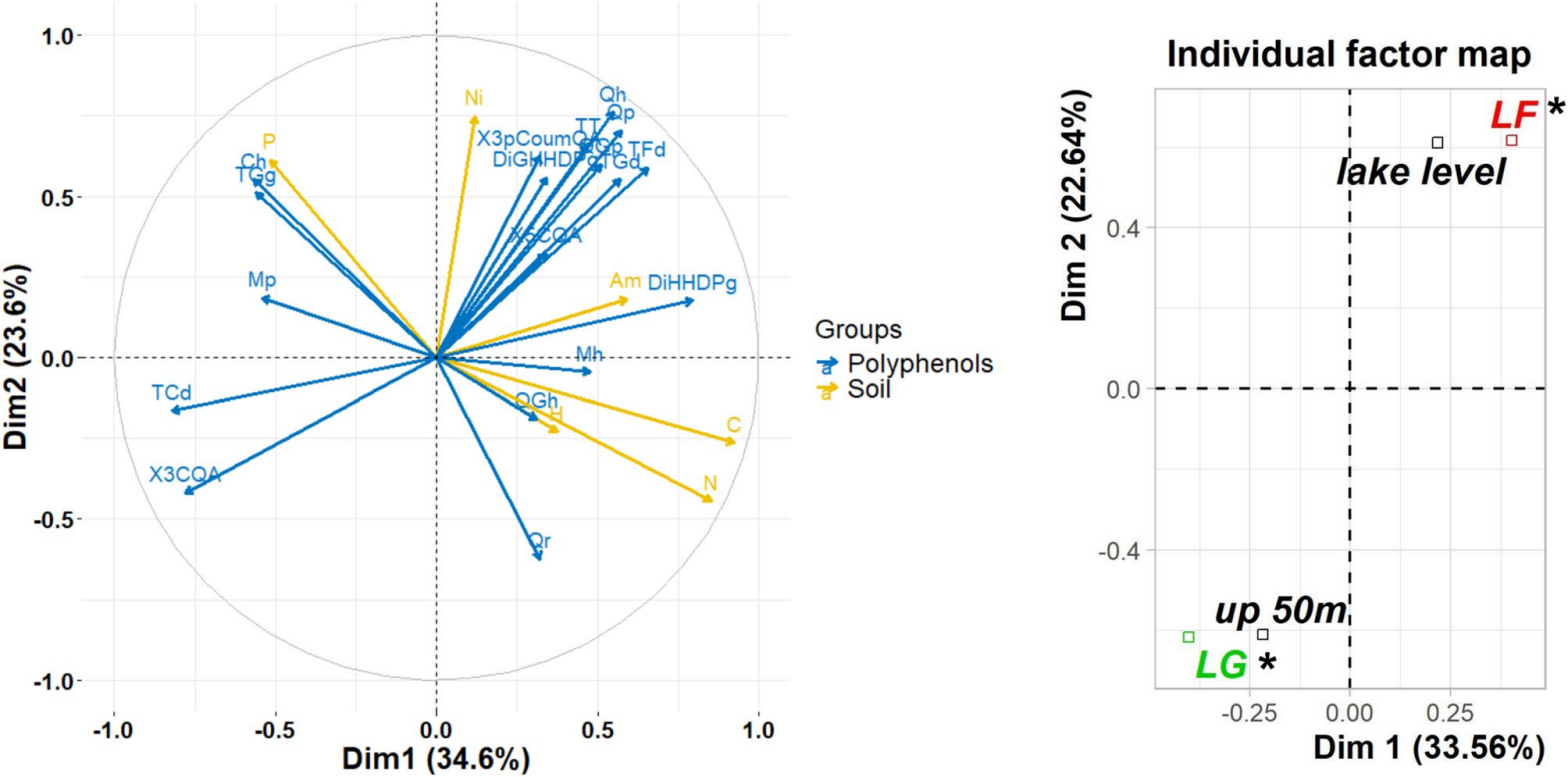
Multiple Factor Analysis (MFA) of the (poly)phenolic concentration data and soil variables of each *N. antarctica* forest site. (**a**) Correlation circle: correlation relationship among variables ((poly)phenolics and soils features) that significantly contributed to dimensions 1 and 2; (**b**) Individual factor map: supplementary variables (latitude and altitude) contribution. HHDP: hexahydroxydephenic acid. DiGHHDPg: Di-Galloyl-HHDP-glucoside, TGg: Tetragalloyl-glucose, X3CQA: 3-Caffeoyl quinic acid, X3pCoumQA: 3-p-Coumaroyl-QA, X5CQA: 5-Caffeoyl quinic acid, Mp: Myricetin-pentoside, QGh: Quercetin-Galloyl-hexoside, Qh: Quercetin-hexoside, Qp: Quercetin-pentoside, Qr: Quercetine-rhamnoside, Qd: Quinic acid derivatives, CF: Conjugated Flavonoids, P: Phosphorus concentration, Ni: nitrate concentration, Am: ammonium concentration, C: carbon concentration, N: nitrogen concentration, *LF*: populations located at 40˚ 26’S, *LG*: populations located at 41˚ 25’S, *lake level*: populations located at 931 and 830 m a.s.l.; *up 50m*: populations located at 985 and 880 m a.s.l. *Significant contribution of the supplementary variables to each dimension by *v*-test.

The Multiple Factor Analysis showed whether (poly)phenolic compounds were positively or negatively correlated with the measured soil variables, thus providing information on how soil characteristics influence the content of bioactive compounds in Ñire leaves. In this analysis, the first two dimensions (Dim 1 and Dim 2) explained together the 58.20% of the accumulated variance among populations (Fig. 4) and Dim 3 and 4 explained together the 25.60% of the variance (data not shown). The relative contributions of each group of variables (soil features and (poly)phenolics) were similar (~ 50%) in the first four dimensions. Carbon (20.26%) and nitrate (22.76%) concentrations were the variables that contributed the most to dimension 1 and 2, respectively (Supplementary Fig. 2).

Among the supplementary variables, only latitude significantly influenced the distribution map of the individuals. Furthermore, the correlation analysis among soil variables and (poly)phenols showed that some variables were strongly correlated (≥ 0.6 correlation coefficient). Di-HHDP-glucoside was positively correlated with the soil total nitrogen concentration (0.70 correlation coefficient), and the soil carbon content (0.64 correlation coefficient); whereas Tetragalloyl-glucose and Caffeoyl-hexoside strongly and negatively correlated with these soil variables (Supplementary Fig. 3). In addition, total nitrogen concentration correlated positively with Quercetin-rhamnoside (0.7 correlation coefficient), and total carbon content negatively correlated with Quinic acid derivatives ( -0.72 correlation coefficient).

## Discussion

This study showed the (poly)phenolic variability in *N. antarctica* infusions elaborated with leaves (of the same phenological stage) from populations growing under different temperatures and soil features. These findings revealed the existence of metabolic diversity related to the secondary metabolite production in *N. antarctica* forests, probably resulting from the species-local environment interactions. Knowing the differential response of *N. antarctica* forests at the level of secondary metabolism provides some clues to understanding the performance of this species under future climate scenarios. Intraspecific variation in the leaf (poly)phenolic content related to the differences in their home environments has previously been reported in other plant species such as *Medicago minima* L.^27^, *Vaccinium myrtillus* L.^28^, *Fagus sylvatica* L.^29^, and *Quercus ilex* L.^30^ In addition, differences in (poly)phenolic concentrations and other chemical compounds (e.g., free amino acids and caffeine) were found in a large panel of tea cultivars^31^. The authors suggested that differences in flavour and pharmacological properties were related to the variability in biochemical compounds. Several factors could influence the (poly)phenolic content of plants, including genetic background, environmental growing conditions, phenological stage at the harvest, post-harvest processing, and storage^10^. Regarding the environmental factors, several studies have suggested that many (poly)phenols, especially phenolic acids, are directly involved in the response of plants to different stress conditions^32–34^, and thus, (poly)phenolic concentrations in natural plants depend on their local growing environment. For example, the effect of geographical location and plantation altitude on the antioxidant capacity and (poly)phenolic composition of green tea infusions have been previously reported^35–37^. In the present study, we found differences in the growing conditions of the *N. antarctica* forests, and this fact could influence the production of bioactive compounds in the populations studied. Several researches have documented the effect of climatic variables on secondary metabolites in the Northern Hemisphere forests (reviewed by Holopanein et al*.*^38^); however, this type of information is very limited or non-existent in Southern Hemisphere forests.

On the one hand, the (poly)phenolic differences found here were partly explained by the differences in the concentration of quercetin derivatives among the provenances of *N. antarctica* populations. In this study, the quercetin concentration ranged from 38.22 to 25.39 mg per 100 ml of infusions elaborated with 2 g of *N. antarctica* leaves. Therefore, a cup of *N. antarctica* infusions could be considered a rich source of quercetin when it is compared with the twenty quercetin-rich foods mentioned in the USDA database^39^. This finding supports the proposal to consider *N. antarctica* leaves as a valuable non-timber product^22^, which is a relevant strategy to increase the profitability of Patagonian silvopastoral systems. At the population level, the infusions elaborated with the *N. antarctica* leaves from *LF* forests were superior in quercetin content, while the *LG_up 50m* population (located at 41°25ʹS–71°29ʹW and 880 m.a.s.l.) showed the lowest quercetin concentration. Interestingly, *LF* sites had a lower frequency of extreme temperatures (i.e., heat and cold stress) than *LGs*, as well as a higher potential for nitrogen mineralization (i.e., N availability). Soil nutrient content affects the secondary metabolite production in plants, mainly through its effect on the enzyme phenylalanine ammonia lyase (PAL)^34^, which is involved in the first step of the flavonoid biosynthesis pathway. Nitrogen or phosphorus deficiency resulted in increased PAL1 transcript levels in Arabidopsis^40^. Variation in PAL level differentially affects the downstream phenylpropanoid pathway, explaining the different accumulation pattern of each flavonoid compound^34^. Influences of the soil characteristics on the (poly)phenolic contents have been documented in some species such as *Vitis vinifera* L.^41^, *Quercus ilex* L.^30^, and some conifers^42^. In tree species, increasing in soil pH showed a negative trend on phenolic content^30,42^. It has also been observed that black soils with high organic matter and nitrogen content promote high (poly)phenolic content and antioxidant capacity^43^. On the contrary, nitrogen or phosphorus depletion strongly increases the accumulation of anthocyanins and kaempferol in Arabidopsis; however, quercetin accumulation does not show the same pattern^34^. In this study, low soil phosphorus (P) concentrations (i.e. below the critical level for plant P availability) were observed in all sites studied. This is not surprising given that Patagonian-Andean forests typically grow on volcanic soils, which are characterised by high organic matter stabilisation, high water-holding capacity, and high P retention (which reduces plant P availability)^44^. Native plants, endemic to these environments, show diverse adaptations to low P availability (e.g. ectomycorrhizae associated with *N. antarctica* forests), which would explain why many species from these temperate forests are not P limited^45,46^. Regarding the total amount of soil carbon, all sites had high levels of this nutrient at the time of the study (soils with more than 3% carbon are considered to have high carbon content^47^). In addition, the negative effect of heat stress on the total phenolic and flavonoid content has been previously reported in other species such as in sugarcane^48^ and European aspen^49^. In the same way, all southern beeches (such as *N. antarctica*) are more adapted to cold temperate conditions and thus, information about secondary metabolism response to heat stress have become relevant in the context of climate change. This study showed that quercetin accumulation does not seem to be favoured by heat stress, considering that the *N. antarctica* leaves were harvested at the end of the hottest month. Notwithstanding the above mentioned, several studies have suggested that the effects on the production of plant secondary metabolites when several stressors occur simultaneously may be greater or even different than those of a single factor^11,50^.

On the other hand, the studied *N. antarctica* infusions are valuable sources of the Caffeoyl-quinic acid derivatives (such as Chlorogenic acid), which ranged from 11.21 (± 0.26) to 17.42 (± 0.06) mg per 100 ml of aqueous extract. At the population level, *LG_up 50m*, which experienced higher frequencies of heat and cold stress and maximum median temperatures at the time of leaf harvest, had the highest amount of 3-Caffeoyl quinic acid (i.e. 3-CQA or Chlorogenic acid). Previous studies have suggested that 3-CQA confers thermotolerance to plants (reviewed by Soviguidi et al*.*^51^), as evidenced by the 3-CQA at temperatures above the species damaging threshold^52^. Nevertheless, no differences in the total amount of 5-Caffeoyl-quinic acid were found among the *N. antarctica* infusions studied. Regarding the minor compounds, some of these (poly)phenols also contributed to the population variability found, especially Caffeoyl-hexoside, TetraGalloyl-glucoside, and Di-Galloyl-HHDP-glucoside. Although the concentrations of these compounds were less than 5 mg per 100 ml of aqueous extract, their influence on the overall quality of *N. antarctica* infusions should not be disregarded, because they also present bioactivity. Interestingly, our results showed that soil nitrogen and carbon concentrations were significantly correlated (positively or negatively) with the concentrations of most of the minor constituents. In addition, *LF* (northern) populations showed higher amounts of Gallic acid derivatives than southern populations (*LGs*). Differences in the concentration of gallic acid have previously been reported for different origins of green tea^36^, ranging from 1.66 (± 0.08) mg in 100 ml of South Korean green tea and 10.60 (± 2.37) mg in 100 ml of Sri Lankan green tea. All these findings underline the need to carry out an extensive biochemical characterization of species-germplasm in order to identify outstanding cultivars or genotypes as a source of superior food and pharmacological products.

## Conclusions

This study showed that infusions prepared with leaves from different *N. antarctica* forests of northern Patagonia are relevant sources of quercetin and cinnamic acid derivatives. Furthermore, the existence of differences in (poly)phenolic concentration among *N. antarctica* infusions depending on the origin of the raw material was revealed. The obtained information is useful for identifying the outstanding *N. antarctica* populations that provide superior non-timber products for the food industry. In addition, our results are relevant for defining forest management strategies, taking into account both the profitability of the system and the conservation of native resources in the Patagonian region.

## Materials and methods

### Plant material and site characterization

Leaves from 10 trees of *N. antarctica* from four different populations in northern Patagonia, Argentina, were randomly collected using a transect collection method, preserving the individuality of each tree (40 samples in total). Harvesting was carried out at the same time of the year (end of January 2021) in all sites, when the leaves were at the fully expanded development stage with an intense green colour. The sampled populations represented pristine and undisturbed forests and included two different latitudinal locations, corresponding to the Falkner Lake (LF: 40°26′S) and Guillelmo Lake (LG: 41°25′S) areas, and two altitudes within them. The environmental conditions for each population studied are summarised in Table 1. Air temperature was recorded by HOBOware^®^ Lite (Massachusetts, USA) every 30 min during two growing seasons (September 2020 to August 2022). Soil samples at a depth of 0–10 cm were collected in triplicate from each site, considering three equidistant points over a transect. In the laboratory, soil samples were sieved through a 2 mm sieve and air dried to measure pH in water (1:2.5), electrical conductivity (1:5 soil:water) and extractable phosphorus (P) content using the ascorbic acid molybdate method^53^. Total carbon (C) and nitrogen (N) were determined by dry combustion (Thermo Electron, FlashEA 1112) following the method describe by Nelson and Sommers^54^^.^ For the determination of ammonium and nitrate, soil samples were extracted with KCl (2 M, 1:10 soil:solution) and analysed for N-NH_4_^+^ and N-NO_3_^−^ using the indophenol blue method and coppered Cd reduction, respectively^55^. For the potential N mineralization test, 100 g soil samples were placed in 0.25 L plastic jars and incubated aerobically for 16 weeks at field capacity (determined as the water content retained in the soil after excess water has been drained away (48 h)) and 25 °C without light. Net potential N mineralization was estimated as total inorganic N (N-NH_4_^+^  + N-NO_3_^−^) at each sampling date (tx) minus the initial concentration at time zero (t0). Four samples per treatment were randomly selected for the determination of inorganic N by a destructive sampling procedure at 0, 2, 8, 12 and 16 weeks. Gravimetric soil water content was estimated to express variables on a dry basis.

### Bioactive compound extraction and quantification

The harvested leaves were freeze-dried, ground to a fine powder using liquid nitrogen, and then used to prepare the aqueous extract. The infusions were prepared according to the method described by Mattera et al.^22^, by adding 100 ml of simmering water (90 ± 2 °C) to 2 g of the dried material and infusing for 5 min with occasional stirring. Infusions were made in triplicate per *N. antarctica* population. All aqueous extracts were carefully filtered prior to chromatographic analysis. For (poly) phenolics quantification, 20 µl of each sample were injected into a reversed-phase HPLC–DAD Agilent 1100 system with a Luna C18 separation column (25 cm × 0.46 cm, 5 µm particle size; Phenomenex, Macclesfield; UK). The mobile phase consisted in a mixture of (A) water: formic acid (99:1 v/v) and (B) acetonitrile; and a flow rate of 0.8 ml × 1 m^−1^ in a linear gradient: 0 min., 5% B; 30 min. 30% B; 40 min. 40% B; 45 min. 60% B; 47 min. 95% B, and after 5 min. of washing the column, return to initial conditions. Compound identification was performed according to the characterization of *N. antarctica* infusions reported by Mattera et al.^22^. Chlorogenic acid, Gallic acid, and Quercetin-rutinoside (Merck KGaA, Darmstadt, Germany) were included as analytical standards, and a calibration curve was established for each one, taking into account eight standards in the concentration range 2–0.031 mM. The identified (poly)phenols were divided into three groups: (1) gallic acid derivatives, caffeic acid and ellagitannins were designated as Gallic acid derivatives (Gd); (2) quinic acid derivatives were designated as Quinic acid derivatives (Qd); (3) esters and glycosides of flavonoids were designated as Conjugated Flavonoids (CF). Three replicates of each infusion were quantified, and the results were expressed as mg per 100 ml aqueous extract.

### Data analysis

Statistical analyses were performed on climate and soil variables to characterise each sampled forest. Normality and homoscedasticity for all variables were first tested by using the Shapiro and Levene tests, respectively. From the temperature datasets of the hottest and coldest months, two subsets were extracted for each population site with the highest and lowest daily temperatures. A non-parametric test was then carried out to assess the existence of differences (p-value < 0.05) between these subsets. In addition, differences in the frequency of days with temperatures above 30 °C and below 0 °C and -5 °C were tested using a Bonferroni test (*p-value* < 0.05). One-way or two-way analysis of variance (ANOVA) was used for soil variables, with ID, latitude, and altitude as main factors. In addition, differences in (poly)phenolic contents among *N. antarctica* infusions were statistically analysed. First, normality and homoscedasticity were tested. If both assumptions were confirmed, a two-way ANOVA followed by the LSD test for multiple comparisons was performed. For those variables where the variance was not homogeneous, a one-way Brown-Forsythe's ANOVA test (B-F_ANOVA) was performed. For variables without a normal distribution, a non-parametric test (Kruskal–Wallis) was chosen. In addition, a principal component analysis (PCA) was performed on the (poly)phenolic concentration data, considering the supplementary variables. The percentage of variance explained by each dimension and the contribution of each (poly)phenolic compound to the first four dimensions were calculated. Then, a Multiple Factor Analysis (MFA) was carried out considering together the (poly)phenolic characterization of each Ñire population and the soil features of the studied sites. Latitude and altitude were considered as supplementary variables in this analysis. The percentage of variance explained by each dimension and the contribution of the groups of variables ((poly)phenols or soil features) to the first four dimensions were calculated. Correlation analyses among all (poly)phenolics and between (poly)phenolics and soil variables were carried out by determining the Spearman coefficients, as it does not assume a normal distribution of the variables, and their significant levels. All data analyses were performed in the R environment^56^. All significant differences were tested at a *p-value* < 0.05.

### Plant material

M.G.M undertook the formal identification of the plant material used in the study. A voucher specimen of this material has not been deposited in a public herbarium. Permission to collect the leaves of *Nothofagus antarctica* in the chosen sites was obtained (authorisation from the Argentinean National Parks Administration).

### Supplementary Information

Supplementary Information.

## Data Availability

The datasets generated during this study are available from the corresponding author on reasonable request.
