# Supplementary material for: Intraspecific variation in leaf (poly)phenolic content of a southern hemisphere beech (Nothofagus antarctica) growing under different environmental conditions
Source: Sci Rep. 2024 Aug 29;14:20050. doi: 10.1038/s41598-024-69939-7 (PMC11362339; doi:10.1038/s41598-024-69939-7)
Supplement: Supplementary file 1 — Supplementary Information. [file 41598_2024_69939_MOESM1_ESM.pdf]

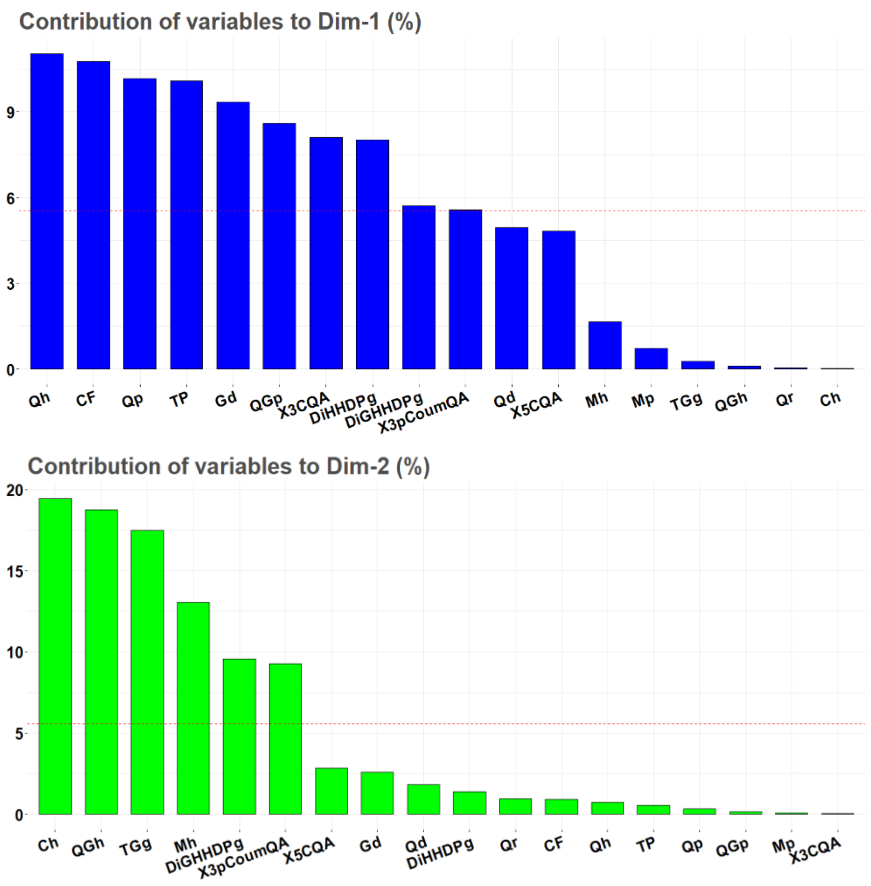

Supplementary Fig. 1 Contribution of (Poly)phenolic compounds to the dimension 1 and 2 of the PCA showed in Fig 2.

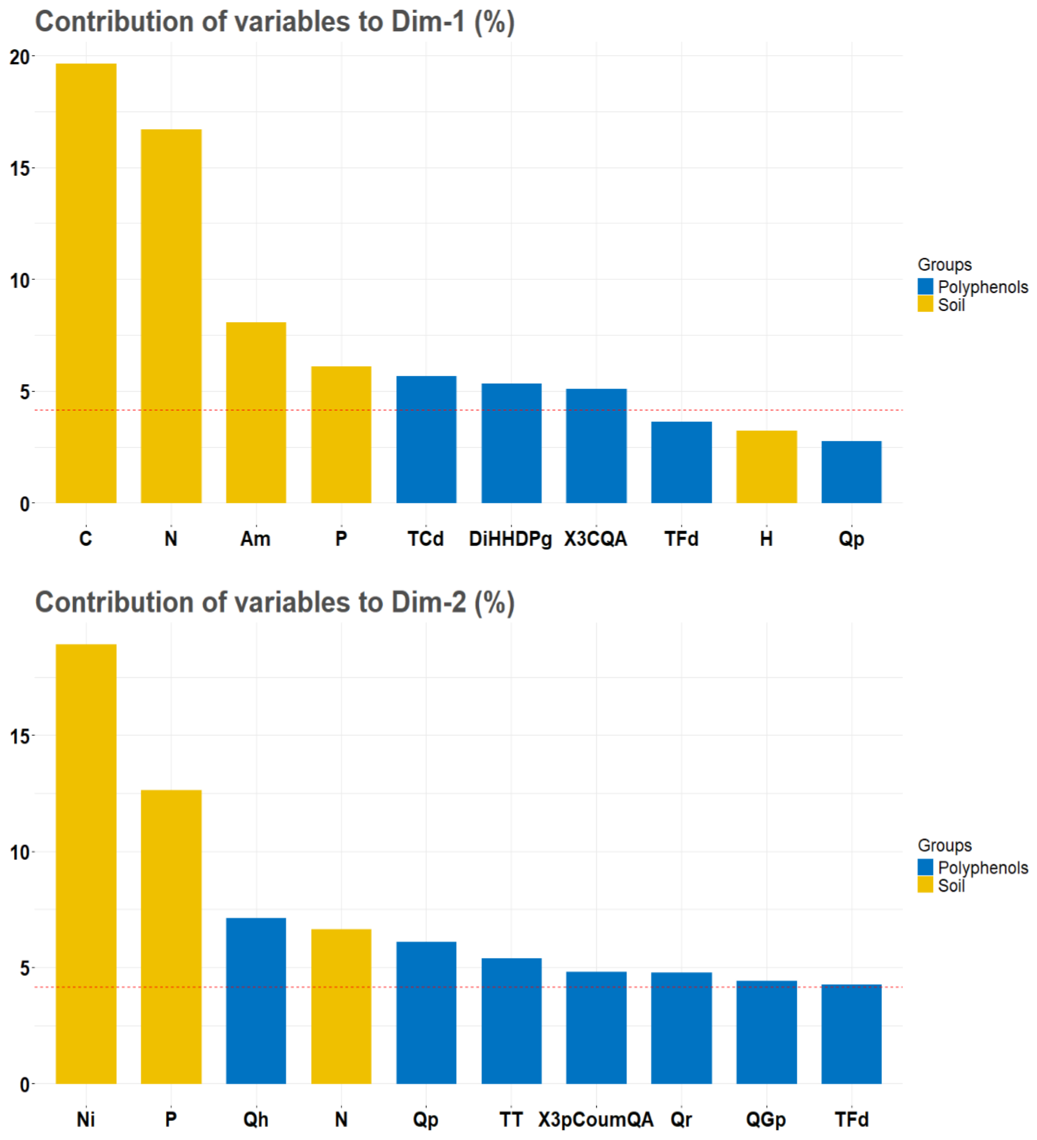

Supplementary Fig. 2 Top ten contribution of Soil variables and (Poly)phenolic compounds to the dimension 1 and 2 of the MFA showed in Fig. 4.

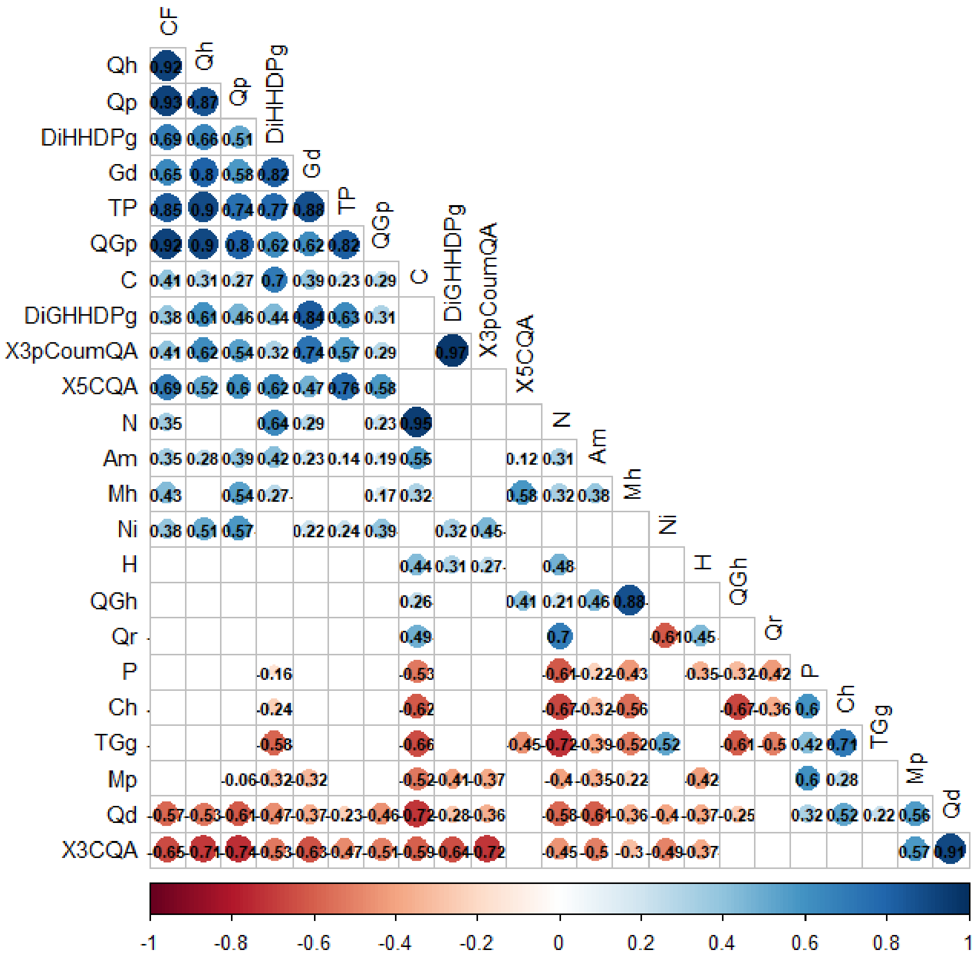

Supplementary Fig. 3 Correlation analysis ("Spearman rank coefficient") between soil variables and (poly)phenolic compounds. Only the values of significantly correlated variables are shown. Abbreviations correspond to the soil variables and (poly)phenolic compounds mentioned in Table 1 and Fig. 1 a, respectively.

Supplementary Table 1. Days with temperatures above 28º C, 30ºC, 32º, and 35ºC during the period between September 2020 and August 2022 in the population sites studied.

ID	Latitude	Longitude	Altitude	Number of days with temperatures:			
				> 28º C	> 30º C	> 32º C	> 35º C
LF_lake level	40˚ 26’ (LF)	71˚ 32’	931(lake level)	32	15 B	2	1
LF_up 50m	40˚ 26’ (LF)	71˚ 32’	985 (up 50m)	45	24 B	8	0
LG_lake level	41˚ 25’ (LG)	71˚ 29’	830 (lake level)	51	30 B	12	1
LG_up 50m	41˚ 25’ (LG)	71˚ 29’	880 (up 50m)	83	58 A	27	8

Statistical analysis was carried out in the yellow column. Bonferroni test was used to assess the existence of differences in the frequency of days with temperatures above 30ºC (number of days with temperatures > 30ºC / total number of days recorded = 729) among the *N. antarctica* population sites. Different letters indicate the existence of significant differences (*p-value* < 0.05).

Supplementary Table 2. Days with temperatures below 0º C, -2ºC, and -5ºC during the period between September 2020 and August 2022 in the population sites studied.

ID	Latitude	Longitude	Altitude	Number of days with temperatures:		
				**< 0º C**	**< -2º C**	**< -5º C**
LF_lake level	40˚ 26’ (LF)	71˚ 32’	931(lake level)	168 B	15 B	8 B
LF_up 50m	40˚ 26’ (LF)	71˚ 32’	985 (up 50m)	163 B	24 B	6 B
LG_lake level	41˚ 25’ (LG)	71˚ 29’	830 (lake level)	259 A	30 B	46 A
LG_up 50m	41˚ 25’ (LG)	71˚ 29’	880 (up 50m)	220 A	58 A	23 A

Statistical analysis was carried out in the yellow columns. Bonferroni test was used to assess the existences of differences in the frequency of days with temperatures below 0 ºC and -5º C (number of days with temperatures < 0º C or -5º C / total of registered days= 729) among the *N. antarctica* population sites. Different letters indicate the existence of significant differences (*p-value* < 0.05).

Supplementary Table 3. Daily thermal range (º C) in the hottest and coldest months of the September 2020 - August 2022.

ID	Latitude	Longitude	Altitude	Daily thermic range (º C) in the hottest month	Daily thermic range (º C) in the coldest month
LF_lake level	40˚ 26’ (LF)	71˚ 32’	931(lake level)	15.95 B	8.22 A
LF_up 50m	40˚ 26’ (LF)	71˚ 32’	985 (up 50m)	16.55 B	7.51 A
LG_lake level	41˚ 25’ (LG)	71˚ 29’	830 (lake level)	20.11 B	9.58 A
LG_up 50m	41˚ 25’ (LG)	71˚ 29’	880 (up 50m)	22.41 A	9.09 A

Statistical analyses are shown in the yellow columns. A non-parametric test was used to assess the existence of differences in the daily thermal range of the hottest and coldest months among the *N. antarctica* population sites. Different letters indicate the existence of significant differences (*p-value* < 0.05).
